# Seminal Plasma Proteomic Biomarkers of Oxidative Stress

**DOI:** 10.3390/ijms21239113

**Published:** 2020-11-30

**Authors:** Rossella Cannarella, Andrea Crafa, Federica Barbagallo, Laura M. Mongioì, Rosita A. Condorelli, Antonio Aversa, Aldo E. Calogero, Sandro La Vignera

**Affiliations:** 1 Department of Clinical and Experimental Medicine, University of Catania, 95123 Catania, Italy; crafa.andrea@outlook.it (A.C.); federica.barbagallo11@gmail.com (F.B.); lauramongioi@hotmail.it (L.M.M.); rosita.condorelli@unict.it (R.A.C.); acaloger@unict.it (A.E.C.); sandrolavignera@unict.it (S.L.V.); 2 Department of Experimental and Clinical Medicine, University Magna Græcia of Catanzaro, 88100 Catanzaro, Italy; aversa@unicz.it

**Keywords:** seminal plasma, proteome, oxidative stress, reactive oxygen species, male infertility

## Abstract

The prevalence of idiopathic male infertility is high, up to 75% of patients with abnormal sperm parameters. Hence, the research of its causes is mandatory. Oxidative stress (OS) can be responsible for male infertility in 30–80% of cases. In recent years, seminal plasma (SP) proteomics has developed as a useful tool to provide biomarkers of specific diseases. This systematic review aims to collect the available evidence on the changes of SP proteome in patients exposed to OS to provide possible SP biomarkers of sperm OS. To accomplish this, the following keyterms “seminal fluid proteome”, “seminal plasma proteome”, “oxidative stress”, and “sperm oxidative stress” were used and 137 records were found. Among these, 17 were finally included. Nine proteins involved with OS were found overexpressed in patients with OS. Twenty-three proteins were found differentially expressed in patients with clinical conditions associated with OS, such as varicocele, male accessory gland infection/inflammation, cigarette smoke, and obesity. These proteins do not seem to overlap among the clinical conditions taken into account. We speculate that specific SP proteins may mediate OS in different clinical conditions. Altogether, these results suggest that proteomics could help to better understand some of the molecular mechanisms involved in the pathogenesis of infertility. However, further studies are needed to identify potential biomarkers of male infertility with valuable clinical significance.

## 1. Introduction

Infertility is a widespread condition in Western Countries. It is defined as the failure to achieve full-term pregnancy after up to 2-year-long unprotected sexual intercourse (WHO, 1983) [1]. In industrialized countries, approximately 15% of couples of childbearing age are infertile [2]. A male factor is responsible for couple infertility in about half of the cases [2]. Some cohort studies have reported that the cause of male infertility remains elusive in up to 75% of the patients with abnormal sperm parameters [3,4]. The search of the causes is, therefore, of paramount importance to plan a targeted therapy [5]. 

Among the etiology of male infertility, oxidative stress (OS) certainly plays a pivotal role. About 30–80% of cases of male infertility can be attributed to sperm damage mediated by an over-production of reactive oxygen species (ROS) [6,7,8,9,10,11]. At physiological levels, ROS participate in sperm functions by promoting sperm capacitation, acrosome reaction, and contributing to sperm motility, thus leading to the fertilization process. However, when there is an imbalance between the production of ROS and the antioxidant capacity, OS occurs, and this impairs male fertility. Specifically, OS decreases male fertility by two main mechanisms:

1. OS damages the sperm cytoplasmic membrane, which is rich in phospholipids with (poly)unsaturated fatty acyl residues highly susceptible to radical damage. This leads to an alteration of sperm motility [12] and its ability to fertilize the oocytes.
2. The excess of radicals causes sperm DNA fragmentation (SDF), resulting in impairment of the paternal genetic contribution to the embryo development [13].

In recent years, proteomics has rapidly and significantly developed, enabling the identification of disease-specific biomarkers. The term “proteomics” refers to the large-scale identification of the protein content of a cell, tissue, or biological fluid [14]. Seminal plasma (SP) consists of secretions originating from the seminal vesicles (65%), prostate (25%), epididymis, and testis (10%) [15]. It plays a crucial role in sperm survival and quality. More specifically, SP influences the female reproductive environment and immunity [16], allows the acquisition of sperm competence [17], and influences the RNA content of spermatozoa and even the embryo development, as transcriptome studies have revealed [18].

Proteomic analysis of SP has recently been considered a valid means to clarify the etiology of male infertility [19,20]. Furthermore, some SP proteins appear to be able to predict sperm recovery in patients with non-obstructive azoospermia or assisted reproductive technique outcome (e.g.,extracellular matrix protein 1 (ECM1), testis-expressed sequence 101 (TEX101), lectingalactoside-binding and soluble 3 binding protein (LGALS3BP) and, therefore, the SP proteome can help in the decision-making during the diagnosis or treatment, as reviewed in Cannarella et al., 2020 [21].

The study of the SP proteome could provide biomarkers of OS in sperm. In fact, the oxidation process induced by free radicals can alter the SP proteome [22]. Oxidized proteins show an increase in carbonyl groups which make them more susceptible to proteolysis. Hence, overexpression of carbonyl proteins could represent a link between OS, alterations in SP proteomics, and infertility [22]. Furthermore, Piomboni and colleagues demonstrated that radicals oxidize the thiol groups of proteins and consequently decrease the overall thiol-disulfide state [23]. Consequently, the loss of free thiol groups could represent another marker of OS [23]. Furthermore, the redox state of thiols in spermatozoa is also related to the tight packaging of chromatin, which normally protects the genomic DNA carried by the spermatozoa. Thus, the alteration of the thiol redox state is related to improper chromatin packing and subsequent DNA damage [24]. The macrophage migration inhibitory factor protein is physiologically involved in maintaining the redox state of thiol and abnormal levels have been found in the SP of patients with altered sperm parameters [25]. The absence of this protein has been shown in patients with a high ROS production [26].

Several studies have evaluated the expression pattern of proteins in the SP of patients with OS. This systematicreview aims to collect the available evidence on the changes of SP proteome in patients exposed to OS to provide possible SP biomarkers of sperm OS. 

## 2. Methods

### 2.1. Sources

The search strategy involved the following databases: Pubmed, MEDLINE, Cochrane, Academic One Files, Google Scholar, and Scopus, from their inception to September 2020. The following combination of Medical Subjects Headings (MeSH) terms and keywords were used: “seminal fluid proteome”, “seminal plasma proteome”, “oxidative stress”, and “sperm oxidative stress”. Other articles were extracted from the reference lists of the articles found by entering the aforementioned keywords.

### 2.2. Study Selection

The study included all the articles that evaluated differences in expression of SP proteome in infertile patients with increased OS. Reviews, comments, letters to the editors, and animal studies on SP proteomics were not included. 

## 3. Results

Thelibrary search resulted in the identification of 137 records. Among these, 43 were duplicates and 25 were not pertinent to the aim of this systematic review. Fifty-two papers were excluded because of the study design, the patients enrolled, or the study outcome. Seventeen articles were finally included [22,26,27,28,29,30,31,32,33,34,35,36,37,38,39,40,41]. These articles were then classified into those investigating SP proteome in patients with an increased OS [22,26,27,34,37,41] and thoseinvestigating SP proteome in patientswith diseasestypicallyassociated with increased OS (e.g., varicocele, male accessoryglandinfection/inflammation (MAGI), smoke, obesity) [28,29,30,31,32,33,35,36,38,39,40]. 

## 4. Seminal Plasma Proteome in Patients with Increased Oxidative Stress

Several proteins have been suggested as possible markers of OS damage. Wang and colleagues, for example, proposed the reduction of DJ-1, a protein secreted from testis, epididymis, and prostate, and involved in counteracting the OS induced by endocrine disruptors, as a possible marker of OS in asthenozoospermic patients [41]. Herwig and colleagues found that the expression of tubulin folding cofactor β, and the increased levels of α-1 chymotrypsin and aldose reductase, are linked to the presence of OS in patients with idiopathic oligo-astheno-teratozoospermia (Herwig et al., 2013) [37]. Another study highlighted that the increased expression of the prolactin-induced protein was also related to OS damage and poor sperm quality [26]. Intasqui and colleagues found overexpression of mucin 5B in normozoospermicmen with high seminal lipid peroxidation levels, suggesting that this protein, produced by bulbourethral glands and involved in the modulation of sperm transport in the male and female tracts, might be a biomarker of lipid peroxidation and thus of OS [22]. However, OS-induced alteration of SP proteome does not occur exclusively in infertile patients, but also in fertile men. Indeed, a recent study has shown that fertile patients with increased OS also have overexpression of proteins involved in stress response such as haptoglobin (HP), peroxiredoxin 4 (PRDX4), and protein S100 calcium binding protein A9 (S100A9). In particular, HP and PRDX4 display antioxidant effects, thus their overexpression is probably an attempt to counteract the effects of ROS overproduction. Instead, the S100A9 protein has a pro-inflammatory effect and its overexpression, together with that of the C3 complement, represents an index of the inflammatory state induced by OS [34].

The SP proteins overexpressed in patients with increased OS and their functions are reported in Table 1.

## 5. Seminal Plasma Proteome in Patients with Diseases Typically Associated with Increased OS

Risky lifestyles and some diseases can increase seminal OS. Indeed, obesity, alcohol abuse, and cigarette smoking are strictly related to OS. Environmental factors, such as exposure to heavy metals, also play a role in ROS overproduction. Other sources of ROS are due to diseases such as male accessory gland infection/inflammation (MAGI) and varicocele [13]. To date, except for varicocele, there are still few studies that have evaluated the alterations of the SP proteome in these groups of patients. Furthermore, there are no studies on the differential protein expression between those with or without increased ROS. Among the limitedavailable evidence, some proteins have been proposed as disease biomarkers. For example, the overexpression of intelectin 1, an infection-induced antimicrobial protein, has been found in asthenozoospermic patients with OS, thus suggesting the possible presence of genital tract infection in these patients. The same study also reported overexpression of alcohol dehydrogenase, involved in alcohol metabolism and aminolevulenic acid dehydratase, increased in case of exposure to heavy metals such as lead. This evidence confirms the role of lifestyle and environmental pollutionin spermdamage and quality impairment by free radicals [41]. 

### 5.1. Varicocele, Oxidative Stress, and Seminal Plasma Proteome

Varicocele is a widespread disease that affects about 15% of men and is responsible for about 40% of primary and 80% of secondary infertility. Varicocele has negative effects on both conventional and bio-functional sperm parameters. Patients with varicocele have lower sperm concentration, total sperm count, progressively motile spermatozoa, and normal forms compared with healthy fertile men, as reviewed in [42]. Additionally, increased percentage of sperm with SDF, mitochondrial dysfunction, and increased externalization of phosphatidylserine (an early sign of apoptosis) occur in patients with varicocele [43].

Increased OS and SDF can be listed among the main mechanisms by which varicocele damages sperm parameters and function. In particular, the loss of testicular thermoregulation in patients with varicocele causes chronic hypoxia and consequent overproduction of ROS. The damage to fertility increases with the worsening of the varicocele degree and is higher when varicocele is bilateral [39].

Several studies have investigated the alterations of the SP proteome in patients with varicocele [31,33,39,44]. Recently, a study aimed to analyze the differential expression pattern between unilateral and bilateral varicoceles has shown that the lower expression of fibronectin (FN1) and suppressor of morphogenesis in the genitalia 1 (SMG1) in patients with bilateral varicocele may be regarded as possible biomarkers to differentiate unilateral from bilateral varicoceles [39].Furthermore, in this study, the main proteins, markers of the damage caused by OS in patients with bilateral varicocele, are aldose reductase, annexin 1, and peroxiredoxin 2. Also, semenogenil (SEMG1) overexpression has been related to ROS and lipid peroxidation in patients with varicocele [44].

Seminal fluid proteomic analysis has shown that insulin-like growth factor-binding protein 7 (IBP-7) and Ig gamma-3 chain C region (HDC) are increased in adolescents with varicocele and normal sperm parameters, whereas the cysteine-rich secretory protein 3 (CRISP-3) is highly overexpressed in adolescents with varicocele and abnormal sperm parameters (Del Giudice et al., 2016) [32]. IBP-7 is a protein involved in cell adhesion and prostacyclin (PGI2) production, whereas HDC is localized in the extracellular space and participates in antigen binding [32]. CRISP-3 participates in the immune response and is associated with chronic inflammation [45]. Thus, the authors hypothesized that varicocele progressively shifts these adolescents from initial immune response to a final chronic inflammatory profile, following by decreased sperm quality [32]. The chronic inflammation leads to ROS overproduction and this supports the hypothesis that seminal alterations may be caused by unbalanced levels of pro-inflammatory cytokines and the scavenger activity. Interestingly, these biomarker proteins may potentially be used to increase the sensibility in establishing the best time when to proceed with varicocele repair [32].

These results were confirmed by another study that reported increased seminal plasma levels of IBP-7, responsible for the proliferative activity, and decreased levels of deoxyribonuclease-1 (DNase1), a protein playing an important role in apoptosis regulation, in adolescents with varicocele of II and III degrees [46]. Specifically, they found that levels of DNase1 were lower in adolescents with varicocele and normal sperm parameters and lowest in adolescents with varicocele and abnormal sperm parameters compared to healthy adolescents without varicocele [46]. The authors supposed that there is a progressive response to varicocele, initially with only an increase of proliferative activity, but when it is followed by a dysregulation of apoptosis, it alters sperm parameters [46].

The main protein associated with SDF in patients with varicocele is fatty acid synthase (FASN), which has been proposed as a marker to differentiate samples with high SDF among patients with varicocele. Another protein typically under-expressed in patients with varicocele is alpha-1-antitrypsin (SERPINA1), involved in the inhibition of proteases implicated in stimulating the inflammatory response. Hence, the under-expression of the protein would indicate a greater inflammatory response in patients with varicocele [39]. Another study by the same group of authors showed that the downregulation of apolipoprotein A2 also appears to be involved in oxidative stress, lipid lipoperoxidation, and SDF in patients with varicocele [40].

The abnormal pattern of SP protein expression in patients with varicocele is further confirmed by their modification after varicocelectomy [31,33]. For example, glyceraldehyde 3 phosphate dehydrogenase (GAPDH) and malate dehydrogenase (MDH) have not been detected in patients with varicocele. These two proteins are involved in the response to OS and in the glycolysis process. The latter is essential for the production of Adenosine triphosphate, which is needed for sperm motility. After varicocelectomy, GAPDHand MDH reappear in the SP and this would confirm the role of the absence of these proteins as biomarkers of OS and impaired sperm motility [31].

In summary, the testicular alterations associated with varicocele modify SP proteome, which, in turn, provides important information on the effects of varicocele and its treatment [33].

### 5.2. Male Accessory Gland Infections, Oxidative Stress, and Seminal Plasma Proteome

The term MAGI indicates a complex nosography entity that includes various clinical manifestations ranging from uncomplicated forms, such as prostatitis, to complicated ones, in which multiple glands may be involved simultaneously up to forms of prostate-vesicular-epididymitis. MAGI negatively affect sperm parameters [47,48]. Indeed, many inflammatory mediators, including ROS and cytokines, have a detrimental effect on germ cells. These mediators often persist even after adequate antimicrobial and anti-inflammatory treatment, indicating dysfunction of the accessory male glands in reconstituting the normal antioxidant capacities of the seminal fluid [47]. The extent of seminal damage caused by MAGI varies according to the number of glands affected. The use of scrotal and transrectal prostate-vesicular ultrasound scans allows to further distinguish MAGI in a congestive hypertrophic form, associated with a better seminal profile than the scleroatrophic variant, albeit with greater production of ROS, probably due to a still active inflammation [47].

To date, very few studies have been carried out to evaluate the modifications of the SP proteome in these diseases. Furthermore, to the best of our knowledge, no study has specifically analyzed the expression of proteins involved in the response to ROS in patients with MAGI. However, the infectious factor and local tissue damage can lead to the infiltration of leukocytes in the inflamed site. This leads to ROS production. The latter triggers immune responses directed against the infectious agent, and the simultaneous secretion of numerous proteases and pro-inflammatory cytokines that increase inflammation. Accordingly, the increased expression of inflammatory proteins in the seminal fluid of patients with MAGI could be related to the increase in OS present in these conditions. Indeed, the expression of some proteins involved in OS response, such as alpha-1 antitrypsin, cystatin proteases, and superoxide dismutase 3 (SOD3), have also been found altered in patients with prostatitis, confirming the role of free radicals in the inflammatory diseases of the genitourinary tract [38]. In particular, the overexpression in prostatitis of type 2 cystatin proteases with anti-inflammatory action, such as cystatin S, cystatin C, cystatin M, cystatin SA, and other protease inhibitors such as alpha-1 antitrypsin in prostatitis, could be regarded as a mechanism to prevent excessive tissue destruction. On the other hand, the reduced expression of SOD3 could indicate a dysfunctional response to OS and inflammation in these patients [38]. Accordingly, several studies have shown that the reduced SOD activity is related to alteration of the sperm morphology, low sperm motility and concentration, an increase in the percentage of dead spermatozoa, and increased DNA fragmentation [49,50,51]. Therefore, protein expression of this and other antioxidative defense enzymes, such as catalase, in seminal plasma might be an additional biomarker of semen quality [52].

In another study conducted on 10 patients with prostate-vesiculo-epididymitis caused by the *Enterococcus faecalis*, the authors showed the exclusive expression of 8 proteins involved mainly in activating the immune system, in patients with infection compared to healthy controls. They also showed a significant difference in expression in 3 proteins related mainly to inflammation metallopeptidase inhibitor 1 (TIMP-1), whey/four-disulfide core (WFDC) domain protein 2, and carboxypeptidase E), between patients and controls [53]. Considering the close relationship between OS and inflammation, it could be useful to evaluate whether the overexpression of proteins is associated with inflammation and increased ROS production.

### 5.3. Cigarette Smoking, Oxidative Stress, and Seminal Plasma Proteome

Cigarette smoke is a major lifestyle factor involved in human diseases. Smoking is associated with an impairment of fertility in both genders. In men, cigarette smoke alters sperm concentration, motility, morphology, and, above all, increases SDF and lipid peroxidation [54]. Exposure of spermatozoa obtained from healthy men with normal sperm parameters to graded concentrations of cigarette smoke extract (CSE) resulted in sperm apoptosis, suggested by the increased number of spermatozoa with the externalization of phosphatidylserine and with fragmented DNA (a sign of late apoptosis) [54]. Moreover, CSE increased the number of spermatozoa with low mitochondrial membrane potential (MMP), a mitochondrial dysfunction that can jeopardize their main energy source, as reviewed in [12]. Interestingly, the detrimental effects of nicotine, the main component of cigarette smoke, detectable in the seminal plasma of cigarette smokers [55], were observed at very low concentrations on SDF [56].The effects of nicotine on sperm function are mediated by interaction with a specific nicotinic receptor (nAChR). We showed that, though 8 nAChR subunits mRNA are expressed in spermatozoa (α1, α3, α4, α6, α7, β2, β4, and δ), only α7 subunit is translated [57]. Furthermore, cotinine, the main metabolite of nicotine, has been experimentally proven to exert detrimental effects on human sperm motility, membrane function, and the ability to undergo capacitation [58].

Increased OS is the main mechanism through which smoking damages spermatozoa. In fact, the substances contained in cigarette smoke, such as cadmium and nicotine, are capable of generating free radicals. The role of OS in smokers is confirmed by the overexpression of the S100A9 protein that, as mentioned above, has been found overexpressed in patients with an increased ROS production [28]. S100A9 belongs to the group of calcium-binding proteins that have the EF-hand domain and binds to pro-inflammatory receptors to start the inflammatory cascade) [59]. Thus, smoking seems to promote an inflammatory response in the sex glands and testis, which in turn leads to impairment of sperm quality [29]. In another study performed on patients with varicocele who smoked, Fariello and colleagues identified five proteins related to apoptosis regulation in moderate smokers (annexin A3 (ANXA3), cathepsin B (CTSB), epididymal secretory protein E3-ß (EDDM3B), prostaglandin-H2 D-isomerase (PTGDS), and extracellular superoxide dismutase (SODE)). Only one protein with a unique expression, the zinc-alpha-2-glycoprotein (ZA2G), was identified in the group of heavy smokers. ZA2G is involved in lipid catabolic process, negative regulation of cell proliferation, and immune response [35]. This study showed that smoking further alters the SP proteome and worsens the OS-induced damage, as proven by the exclusive expression in smoking patients of proteins involved in the inflammatory response and neutralization of free radicals, such as SODE [35].

Moreover, a more recent study showed that the under-expression of two epididymal proteins, neprilysin and ß-defensin 106A, and the overexpression of histone H4A, are capable of predicting the smoker group [28]. Neprilysin is a transmembrane protein, mainly involved in immunoreactivity and antimicrobial defense in the male genital tract. The ß-defensin 106A is specifically expressed in the human epididymis and it is mainly involved in the regulation of the inflammatory response. Therefore, in smokers, the downregulation of this protein can lead to altered epididymal homeostasis [28].

### 5.4. Obesity, Oxidative Stress, and Seminal Plasma Proteome

Very few studies have analyzed the expression pattern of SP proteins of obese men, despite the fact that obesity represents a global public health problem (https://www.who.int/health-topics/obesity#tab=tab_1). About 1.9 billion persons over the age of 18 (39% of the population) were overweight in 2016. Among these, over 650 million (13%) were obese (https://www.who.int/health-topics/obesity#tab=tab_1). Male obesity severely impacts the quality of semen. We have shown that overweight and even more obese men have abnormal conventional sperm parameters and, in particular, asthenozoospermia and teratozoospermia [60]. A meta-analysis of 21 studies, collecting a total sample of 13,077 men, showed that overweight and obesity are also associated with a higher prevalence of azoospermia and oligozoospermia [61]. Moreover, in a previous study, we have shown that the bio-functional sperm parameters are also altered in overweight/obese men. In particular, obese patients showed an increased percentage of spermatozoa with low MMP and a higher percentage of spermatozoa with SDF and phosphatidylserine externalization, an early marker of apoptosis [60]. 

Several mechanisms have been hypothesized to explain obesity-induced sperm damage. These include the alteration of the endocrine hormonal profile and the state of systemic inflammation. The latter is closely correlated with the increase in OS levels that occur in these patients [62]. Indeed, obesity is associated with increased production of pro-inflammatory cytokines, such as interleukin-6 (IL-6) and tumor necrosis factor α (TNFα), leading to a chronic inflammatory state [62]. This disrupts the seminiferous and the epididymal epithelium by an overproduction of ROS. Furthermore, the inflammatory process recalls phagocytic leukocytes that contribute to increase the amount of ROS [63].

A pivotal role is also played by heat-induced damage. In fact, testicular thermal stress increases in obese men mainly due to the deposition of fat in the suprapubic region and around the pampiniform plexus. The resulting increase in testicular temperature decreases sperm motility and concentration, and increases SDF and OS levels [64]. 

The proteomic profile of the seminal plasma of obese men seems to confirm the role played by the OS in impairing fertility in these patients. Indeed, a recent study highlighted the presence of elevated levels of ceruloplasmin, clusterin, glutathione peroxidase, mitochondrial glutathione reductase, and ADP ribosyl cyclase in the SP of obese men [36]. All these proteins are involved in the antioxidant activity, in the cellular response to the superoxide anion, and the detoxification of hydrogen peroxide, counteracting the damage induced by temperature and OS [36]. Their overexpression probably represents an attempt to preserve homeostasis from the damage induced by excessive ROS production [36]. Furthermore, the overexpression of proteins previously mentioned, such as HP and S100A9 that are involved in stress response, has also been reported [36].

## 6. Conclusions

Numerous studies have shown differential protein expression in various clinical conditions. The huge amount of data available does not allow for immediate interpretation. This review summarized the differential protein expression under conditions characterized by increased levels of OS in the seminal fluid (Table 2). These proteins do not appear to overlap in patients with varicocele, MAGI, obesity, and cigarette smokers. It can be hypothesized that specific SP proteins may mediate the effects of OS, although their accuracy has not yet been clearly demonstrated. Further studies are therefore needed to confirm this hypothesis and to better understand the interaction between OS and seminal proteome and to identify potential biomarkers with valuable clinical significance in infertile males with increased OS.

## Figures and Tables

**Table 1:** Seminal plasma proteins overexpressed in patients with increased oxidative stress.

SP protein	Reference	Function
Aldose reductase	[37]	It converts glucose to sorbitol during the polyol pathway of glucose metabolism
α1-chymotrypsin	[37]	It has proteolytic activity against the chymotrypsin-specific substrate N-Succinyl-Ala-Ala-Pro-Phe-p-nitroanilide. It is released by granulocytes.
DJ-1	[41]	DJ-1 activation is catalyzed by ROS. When active, DJ-1 inhibits removal of NFκB signal
Haptoglobin	[34]	It is a late positive acute phase protein of inflammation
Mucin 5B	[22]	It increases the SP viscosity and correlates with inflammation, hypoxia, and OS
Peroxiredoxin 4	[34]	Belongs to a family of peroxide-degrading enzymes, involved in cellular OS control
Prolactin-induced protein	[26]	Extracellular matrix protein that can mediate tissue responses to inflammation
Protein S100A9	[34]	It plays an important role in cell differentiation and OS response
Tubulin folding cofactor β	[37]	It acts in the development of α/β-tubulin heterodimers, which are critical for the normal growth of mammalian cells. It acts in the development of hypoxic-ischemic injury

Abbreviations: NFκB, nuclear factor kappa light chain enhancer; OS, oxidative stress; ROS, reactive oxygen species; SP, seminal plasma; S100A9, S100 calcium binding protein A9.

**Table 2:** Expression pattern and function of proteins involved in the response to ROS in diseases associated with increased oxidative stress.

Reference	Disease	Proteins	Expression Pattern	Function
[39]	Bilateral Varicocele	Aldose reductase	Overexpressed	Responsible for the induction of the sperm capacitation process
		Annexin 1	Overexpressed	Protein with anti-inflammatory properties
		PRDX1	Overexpressed	Involved in response to ROS and OS
		PRDX2	Overexpressed	Involved in response to ROS and OS
		FN1	Under-expressed	Involved in seminal gel formation and stimulates sperm capacitation
		alpha-1 antitrypsin	Under-expressed	Acute-phase protein responsible for the inhibition of proteases involved in stimulating the inflammatory response
[40]	Varicocele	APO A2	Under-expressed	Involved in pathways such as OS response, lipid peroxidation, and SDF
[44]	Varicocele	SEMG1	Overexpressed	Involved in semen coagulation. Its increasing in varicocele may reflect a strategy to counteract ROS and lipid peroxidation
[31]	Varicocele pre-treatment	Clusterin	Overexpressed	Related to preservation of the damage caused by oxidative reactions
	Varicocele post-treatment	DJ-1	Overexpressed	Linked to ROS response
		SOD	Overexpressed	Linked to ROS response
		S100A9	Overexpressed	It plays an important role in cell differentiation and OS response
		GAPDH	Exclusive expression in post-treated patients	Linked to ROS response, NAD-binding function, and gluconeogenesis
		MDH	Exclusive expression in post-treated patients	Linked to ROS response, NAD-binding function, and gluconeogenesis
[38]	MAGI	Cystatin proteases	Overexpressed	Protease inhibitors involved in inflammatory response
		alpha-1 antitrypsin	Overexpressed	Protease inhibitors involved in inflammatory response
		SOD 3	Under-expressed	Linked to ROS response
[29]	Cigarette smoke	S100A9	Overexpressed	It binds pro-inflammatory receptors to initiate the inflammatory cascade
[35]	Cigarette smoke	SODE	Exclusive expression in moderate smokers	Antioxidant role removing superoxide radicals
[36]	Obesity	ADP ribosyl cyclase	Overexpressed	Antioxidant activity, cellular response to superoxide anion, and detoxification of hydrogen peroxide
		Ceruloplasmin,	Overexpressed	Antioxidant activity, cellular response to superoxide anion, and detoxification of hydrogen peroxide
		Glutathione peroxidase	Overexpressed	Antioxidant activity, cellular response to superoxide anion, and detoxification of hydrogen peroxide
		Clusterin	Overexpressed	Antioxidant activity, cellular response to superoxide anion, and detoxification of hydrogen peroxide
		Mitochondrial glutathione reductase	Overexpressed	Antioxidant activity, cellular response to superoxide anion, and detoxification of hydrogen peroxide
		HP	Overexpressed	It is a late positive acute-phase protein of inflammation
		S100A9	Overexpressed	It plays an important role in cell differentiation and OS response

Abbreviations: APOA2, Apolipoprotein A2; FN1, fibronectin; G3P, glyceraldehyde 3 phosphate dehydrogenase; HP, haptoglobin; MAGI, male accessory gland infection/inflammation; MDH, malate dehydrogenase; OS, oxidative stress; PRDX, Peroxyredoxin; ROS, reactive oxygen species; SDF, sperm DNA fragmentation; SEMG1, semenogelin 1; SERPINA 1, α1-antitrypsin; SODE, extracellular superoxide dismutase.
